# The relationship between sex hormones and glycated hemoglobin in a non-diabetic middle-aged and elderly population

**DOI:** 10.1186/s12902-022-01002-w

**Published:** 2022-04-05

**Authors:** Yiting Xu, Weijie Cao, Yun Shen, Junling Tang, Yufei Wang, Xiaojing Ma, Yuqian Bao

**Affiliations:** Department of Endocrinology and Metabolism, Shanghai Jiao Tong University Affiliated Sixth People’s Hospital; Shanghai Clinical Center for Diabetes; Shanghai Key Clinical Center for Metabolic Disease; Shanghai Diabetes Institute; Shanghai Key Laboratory of Diabetes Mellitus, Shanghai, 200233 China

**Keywords:** Glycation, Hemoglobin, Diabetes, Estradiol, Sex hormone-binding globulin

## Abstract

**Background:**

Sex hormones are strongly linked to the occurrence and development of diabetes, and influence glycated hemoglobin (HbA_1c_) levels in diabetic population; but, the relationship between sex hormones and HbA_1c_ in non-diabetic population remains unknown. This study aimed to explore the extent of influence of sex hormones on HbA_1c_ levels in non-diabetic population.

**Methods:**

A total of 1409 non–diabetic subjects, including 601 men and 808 postmenopausal women were recruited from Shanghai community. HbA_1c_ was detected using high performance liquid chromatography, and hemoglobin level was determined by sodium lauryl sulfate colorimetry. Serum estradiol (E_2_), total testosterone (TT), and sex hormone binding globulin (SHBG) were measured by chemiluminescent microparticle immunoassays.

**Results:**

The level of HbA_1c_ was 5.6 (5.4–5.9) % in all subjects, with 5.6 (5.4–5.8) % in men and 5.7 (5.5–5.9) % in postmenopausal women. After adjusting for age, body mass index (BMI), and hemoglobin, E_2_ was positively correlated with HbA_1c_ in men (*r* = 0.122, *P* = .003), and SHBG was inversely correlated with HbA_1c_ (*r* = − 0.125, *P* < .001) in women. Other hormones were not correlated with HbA_1c_ (all *P* > .05). Multivariate linear regression analysis showed that, except for traditional factors, such as age, hemoglobin, and BMI, E_2_ was another determinant of HbA_1c_ (standardized *β* = 0.137, *P* = .003) in men; besides, in women, SHBG was another determinant of HbA_1c_ (standardized *β* = − 0.178, *P* < .001), except for age and systolic blood pressure.

**Conclusion:**

After controlling for confounding factors, two sex hormones, as E_2_ and SHBG could influence HbA_1c_ levels in non-diabetic population.

**Supplementary Information:**

The online version contains supplementary material available at 10.1186/s12902-022-01002-w.

## Introduction

Sex hormones, including estrogen, progesterone, and androgens, are synthesized by the human gonads and adrenal cortex. Their main role is to promote development of the reproductive system and maintain secondary sexual characteristics. More than that, low circulating levels of testosterone and sex-hormone-binding globulin (SHBG) were reported to be associated with increased cardiovascular risk in men, possibly due to effects on insulin resistance and glycemia. Recent studies have found that men with low serum testosterone levels had increased risk of diabetes; however, it was in contrast to that in women [1–3]. Additionally, lower serum total testosterone and sex hormone binding globulin levels were found in patients living with diabetes [4, 5].

HbA_1c_ has long been regarded as the gold standard for evaluating long-term blood glucose control in patients living with diabetes in clinical routines. In recent years, standardized detection of HbA_1c_ has been constantly improved, leading to sufficient sensitivity and specificity for HbA_1c_ as an indicator of diabetes diagnosis [6–8]. But relatively few data are available on the relationship between sex hormones and this marker of long-term glycaemia. One study found that serum testosterone level was positively correlated with that of HbA_1c_ in men with type 2 diabetes mellitus [9]. The association between low SHBG and HbA_1c_ was also found in postmenopausal women; further, low total testosterone (TT) and SHBG were reported to be associated with HbA_1c_ even below the threshold for diabetes in middle-aged and older men [10–12], which suggested that sex hormones might be markers of pathological processes resulting in elevated glucose levels among populations without diabetes. However, there is no evidence to delineate the relationship between the levels of sex hormones and HbA_1c_ in a non-diabetic population. Thus, this study recruited non-diabetic subjects from Shanghai communities to explore whether the association of sex hormones with HbA_1c_ levels is similar in a non-diabetic population to in a diabetic population.

## Materials and methods

### Study population

We recruited the subjects aged 40 years and over who volunteered to participate and were able to provide required information in Shanghai communities between October 2015 and July 2016. Patients living with diabetes were excluded according to prior medical diagnoses or receiving hypoglycemic therapy, or meeting the diagnostic criteria of the 2010 ADA, which states that diabetes is defined by fasting plasma glucose (FPG) ≥ 7.0 mmol/L and/or 2-h plasma glucose (2hPG) ≥ 11.1 mmol/L and/or HbA_1c_ ≥ 6.5% [6]. Other exclusion criteria included a known history of cardiovascular and cerebrovascular diseases, malignant tumors, severe liver or kidney dysfunction, thyroid dysfunction, severe anemia, treatment with steroids or thyroxine or estrogen or androgen, using oral drugs to treat metabolic syndrome and hyperinsulinemia, cystic fibrosis, and nonmenopausal women. Menopause is defined as 12 consecutive months of amenorrhea without other medical behaviors [13]. This study was approved by the Ethics Committee of the Sixth People’s Hospital Affiliated to Shanghai Jiao Tong University. All subjects signed an informed consent form before participation. All non-diabetic subjects received standardized questionnaires, including previous and present illness and treatment, physical examinations, and biochemical determinations.

### Anthropometric and biochemical measurements

Height, weight and blood pressure were measured using the previously standardized method [14]. Body mass index (BMI) = weight (kg) / height (m^2^). Venous blood samples were collected after a 10 h overnight fast to detect fasting blood glucose (FPG), HbA_1c_, fasting insulin (FINS), blood lipids [total cholesterol (TC), triglycerides (TG), high-density lipoprotein cholesterol (HDL-c), and low-density lipoprotein cholesterol (LDL-c)], and C-reactive protein (CRP). Additionally, all the non-diabetic subjects were tested for hemoglobin, and blood samples were collected after 75 g oral glucose tolerance test to determine the 2-h blood glucose (2hPG). All laboratory indicators were measured using the standard methods. The homeostasis model assessment of insulin resistance (HOMA-IR) was as follows: HOMA-IR = FINS (mU/L) × FPG (mmol/L)/22.5 [15].

HbA_1c_ level was measured by high performance liquid chromatography (Variant II hemoglobin analyzer; Bio–Rad, Hercules, CA, USA), and hemoglobin level was measured by sodium lauryl sulfate colorimetry (Sysmex XE–2100 hematology analyzer, Sysmex Corporation, Kobe, Japan). The intra-assay and inter-assay coefficients of variation for HbA_1c_ were < 2.60% and < 3.40%, respectively. Serum insulin levels were measured with an electrochemiluminescence immunoassay on a Cobas e411 analyzer (Roche Diagnostics GmbH, Mannheim, Germany) with intra- and interassay coefficients of variation of 1.7 and 2.5%, respectively. Serum estradiol (E_2_), TT, and SHBG were detected on Abbott Architect i2000SR analyzer by chemiluminescence microparticle immunoassay (kits from Abbott GmbH & Co. KG, Wiesbaden, Germany). Bioactive testosterone (BT) = N × [FT]; Kt = 1 × 10^9^ L/mol, N = Ka × Ca + 1, where Ka = 3.6 × 10^4^ L/mol; and Ca is Alb level. The sensitivity of E_2_, TT, and SHBG estimation was < 2.5 pg/mL, < 0.1 ng/mL, and <  0.3 mmol/L, respectively. The intra-assay and inter-assay coefficients of variation for E_2_ were < 2.40% and < 2.70%, respectively; the intra-assay and inter-assay coefficients of variation for TT were < 2.16% and < 2.23%, respectively. The intra-assay and inter-assay coefficients of variation for SHBG were < 2.80% and < 4.30%, respectively.

### Statistical analyses

The research data was analyzed using SPSS, version 20.0 (SPSS Inc., Chicago, IL, USA). All variables were tested for normality. Normally distributed variables are presented as mean ± standard deviation, and non–normally distributed variables are presented as median and interquartile range. Student’s *t*-test was used to compare two groups with normal distribution, whereas Wilcoxon rank sum test was used for skewed distribution between two groups. Partial correlation analysis was conducted to analyze the potential correlation between the levels of HbA_1c_ and sex hormones. A multivariate linear regression was performed to examine this correlation in men and women, respectively. All *P* values were two-tailed, and *P* < .05 was considered statistically significant.

## Results

### Clinical characteristics of study subjects

**Fig. 1:**
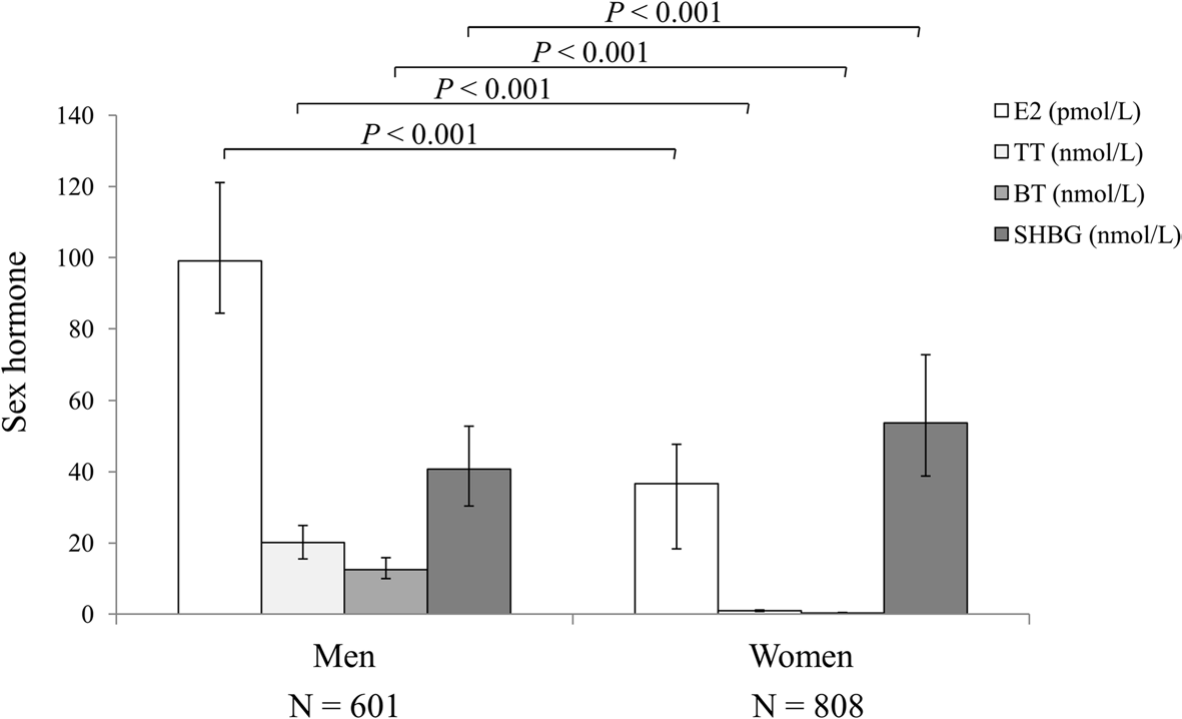
Comparison of sex hormone levels between men and postmenopausal women. The columns represent the median value, and error bars indicate the 25th and 75th percentile

A total of 1409 cases with an average age of 60.4 ± 6.2 years were enrolled in this study, including 601 men, and 808 postmenopausal women with a median menopause duration of 10 (6–15) years. HbA_1c_ level was 5.6 (5.4–5.9) % in all the subjects, with 5.6 (5.4–5.8) % in men and 5.7 (5.5–5.9) % in postmenopausal women. Men had a median E_2_ level of 99.1 (84.4–121.1) pmol/L, median TT of 20.12 (15.5–24.9) nmol/L, median BT of 12.5 (10.0–15.9) nmol/L, and median SHBG of 40.7 (30.4–52.8) nmol/L. Postmenopausal women had a median E_2_ level of 36.7 (18.4–47.7) pmol/L, median TT of 0.9 (0.8–1.2) nmol/L, median BT of is 0.3 (0.2–0.3) nmol/L, and median SHBG of 53.7 (38.8–72.8) nmol/L (Table 1). Levels of E_2_, TT, and BT were significantly higher while those of SHBG were lower in men than in women (all *P* < .001) (Fig. 1). Additionally, the levels of BMI, SBP, DBP, and TG were higher in men than in women (all *P* < .05); whereas, women tended to have higher HbA_1c_, HOMA-IR, TC, HDL-c, and LDL-c levels than that in men (all *P* < .05).

**Table 1:** Characteristic of the study subjects

Variables	Total (*n* = 1409)	Men (*n* = 601)	Women (*n* = 808)
Age (years)	60.4 ± 6.2	60.3 ± 7.2	60.5 ± 5.3
BMI (kg/m^2^)	24.0 ± 3.2	24.5 ± 3.0	23.6 ± 3.3 ^**^
SBP (mmHg)	130.0 (119.0–142.0)	133.0 (123.0–145.0)	127.0 (116.0–139.0) ^**^
DBP (mmHg)	77.0 (71.0–84.0)	80.0 (74.0–87.0)	75.0 (69.0–82.0) ^**^
FPG (mmol/L)	5.7 ± 0.5	5.7 ± 0.5	5.7 ± 0.5
2hPG (mmol/L)	7.0 ± 1.7	7.0 ± 1.8	7.1 ± 1.7
FINS (mU/L)	8.6 (6.2–12.2)	8.3 (5.8–11.6)	8.9 (6.4–12.3) ^*^
HOMA-IR	2.1 (1.5–3.1)	2.1 (1.5–3.0)	2.2 (1.6–3.2) ^*^
TC (mmol/L)	5.4 (4.8–6.0)	5.0 (4.5–5.6)	5.6 (5.0–6.3) ^**^
TG (mmol/L)	1.4 (1.0–2.0)	1.4 (1.0–2.2)	1.3 (1.0–1.9) ^**^
HDL-c (mmol/L)	1.4 (1.2–1.7)	1.3 (1.1–1.5)	1.5 (1.3–1.8) ^**^
LDL-c (mmol/L)	3.3 ± 0.8	3.1 ± 0.8	3.4 ± 0.8 ^**^
CRP (mg/L)	0.9 (0.4–1.6)	0.8 (0.4–1.5)	1.0 (0.5–1.6)
HbA_1c_ (%)	5.6 (5.4–5.9)	5.6 (5.4–5.8)	5.7 (5.5–5.9) ^**^
Hemoglobin (g/L)	144.0 (135.0–154.0)	154.0 (147.5–161.0)	137.0 (132.0–143.0) ^**^
Years since menopause (years)	/	/	10 (6–15)

Continuous variables are expressed as means ± standard deviation or medians with interquartile range. Categorical variables are expressed as numbers with percentages

Men versus Women, ^*^ *P* < .05, ^**^ *P* < .01

*Abbreviation*: *BMI* Body mass index, *SBP* Systolic blood pressure, *DBP* Diastolic blood pressure, *FPG* Fasting plasma glucose, *2hPG* 2-h plasma glucose, *HOMA* ***-*** *IR* Homeostasis model assessment-insulin resistance index, *TC* Total cholesterol, *TG* Triglyceride, *HDL-c* High-density lipoprotein cholesterol, *LDL-c* Low-density lipoprotein cholesterol, *CRP* C-reactive protein, *HbA*_*1c*_ Glycated hemoglobin A_1c_

### Associations between sex hormone levels and HbA_1c_

**Fig. 2:**
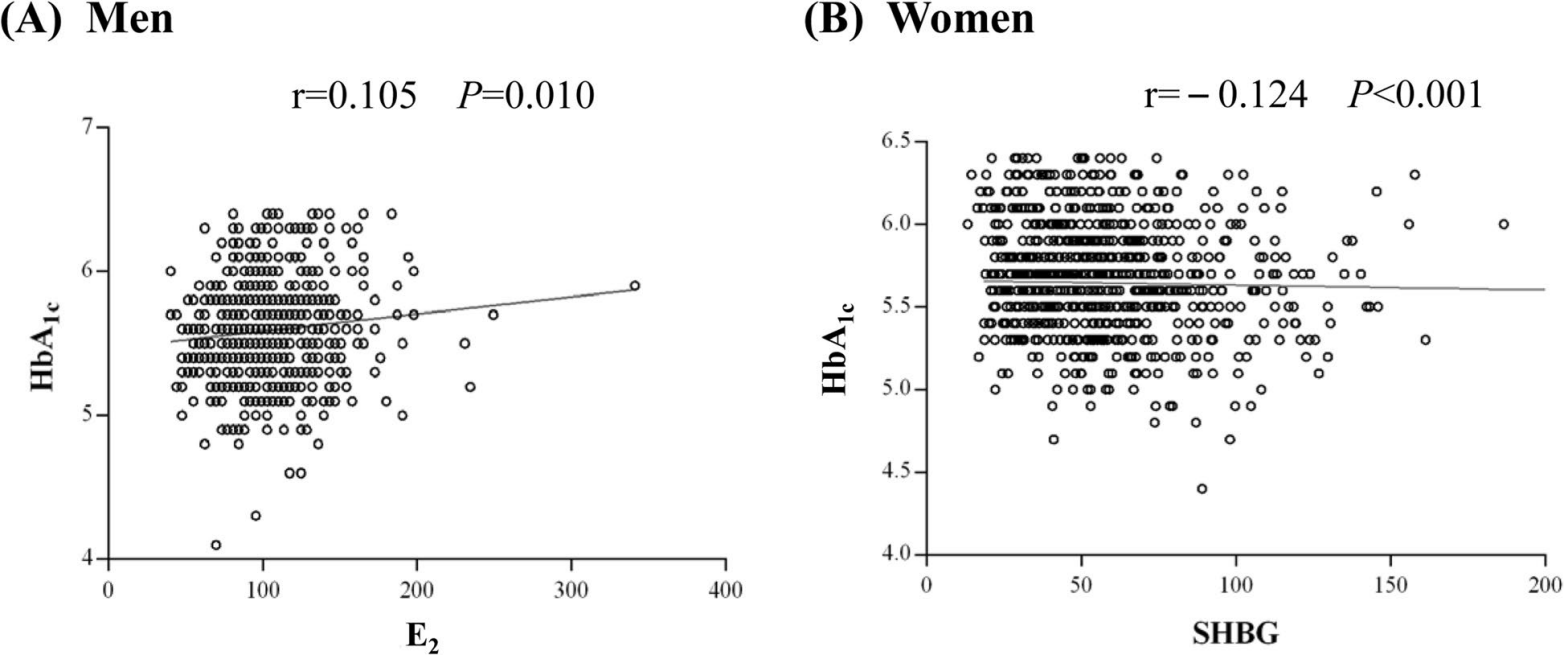
Correlation of E_2_ (in men) and SHBG (in women) with HbA_1c_ levels

Figure 2 showed the correlation of E_2_ with HbA_1c_ in men and SHBG with HbA_1c_ in women. Partial correlation analysis after adjusting for age, BMI, and hemoglobin levels showed that only E_2_ was positively correlated with HbA_1c_ in men (*r* = 0.122, *P* = .003); BT was marginally correlated with HbA_1c_ (*r* = 0.080, *P* = .05). TT and SHBG were not correlated to HbA_1c_ (*P* = .238; *P* = .318). In women, only SHBG was negatively correlated with HbA_1c_ (*r* = − 0.125, *P* < .001), while other hormones were not correlated with HbA_1c_ (all *P* > .05) (Table 2).

**Table 2:** Partial correlations of HbA_1c_ levels with sex hormones

HbA_1c_	Men		Women	
	*r*	*P*	*r*	*P*
E_2_	0.122	0.003	0.007	0.842
TT	0.048	0.238	0.029	0.411
BT	0.080	0.050	0.039	0.274
SHBG	−0.041	0.318	−0.125	< 0.001

Partial correlation analysis adjusted age, BMI and hemoglobin

*Abbreviation: HbA*_*1c*_ Glycated haemoglobin A_1c_, *E*_*2*_ Estradiol, *TT* Total testosterone, *BT* Bioavailable testosterone, *SHBG* Sex hormone-binding globulin, *BMI* Body mass index

### Analysis of sex hormones affecting HbA_1c_

A multivariate linear regression model was constructed with data from men and postmenopausal women to analyze the association of sex hormones with HbA_1c_. In men, age, BMI, SBP, DBP, FPG, 2hPG, HOMA-IR, TC, TG, HDL-c, LDL-c, CRP, hemoglobin, and E_2_ were independent variables, and HbA_1c_ was the dependent variable. We found that except for hemoglobin and BMI, E_2_ was a positive factor for HbA_1c_ (standardized *β* = 0.137, *P* = .003) (Table 3). Further, in postmenopausal women, age, BMI, SBP, DBP, FPG, 2hPG, HOMA-IR, TC, TG, HDL-c, LDL-c, CRP, hemoglobin, and SHBG were independent variables, and HbA_1c_ was the dependent variable. Except for age and SBP, SHBG was a negative factor for HbA_1c_ (standardized *β* = − 0.178, *P* < .001).

**Table 3:** Multivariate regression analysis on HbA_1c_ in men and women

HbA_1c_	Multivariate model		
	standardized *β*	t	*P*
Men (*n* = 601)			
hemoglobin	− 0.162	− 3.925	< 0.001
FPG	0.165	4.166	< 0.001
E_2_	0.134	3.384	0.001
BMI	0.136	3.415	0.001
age	0.115	2.868	0.004
LDL-c	0.087	2.200	0.028
Women (*n* = 808)			
SHBG	−0.161	−3.858	< 0.001
age	0.200	3.505	< 0.001
FPG	0.129	3.277	0.001
SBP	0.114	2.354	0.019

For men, multivariate model included age, BMI, SBP, DBP, FPG, 2hPG, HOMA-IR, TC, TG, HDL-c, LDL-c, CRP, hemoglobin and E_2_. For women, multivariate model included age, years since menopause, BMI, SBP, DBP, FPG, 2hPG, HOMA-IR, TC, TG, HDL-c, LDL-c, CRP, hemoglobin and SHBG

*Abbreviation*: *HbA*_*1c*_ Glycated haemoglobin A_1c_, *E*_*2*_ Estradiol, *BMI* Body mass index, *SHBG* Sex hormone-binding globulin, *SBP* Systolic blood pressure, *DBP* Diastolic blood pressure, *FPG* Fasting plasma glucose, *2hPG* 2-h plasma glucose, *HOMA-IR* Homeostasis model assessment-insulin resistance index, *TC* Total cholesterol, *TG* Triglyceride, *HDL-c* High-density lipoprotein cholesterol, *LDL-c* Low-density lipoprotein cholesterol, *CRP* C-reactive protein

## Discussion

To our knowledge, the present study was the first to assess the association between a relatively complete set of sex hormones and HbA_1c_, and was conducted in both men and postmenopausal women. We found that E_2_ was a positive factor for HbA_1c_ in men while SHBG was a negative factor for HbA_1c_ in postmenopausal women.

At a cellular level, testosterone increases the expression of insulin receptor β subunit, insulin receptor substrate-1, protein kinase B and glucose transporter type 4 in adipose tissue and adenosine 5′-monophosphate-activated protein kinase expression and activity in skeletal muscle [16, 17]. Hence, it was reported that testosterone enhances insulin sensitivity in obese men with hypogonadism by decreasing fat mass, increasing lean mass, decreasing free fatty acids and suppressing inflammation [18]. In addition, androgen therapy increases hemoglobin concentration and stimulates erythropoiesis [19]. Some studies suggest that low BT can compromise erythropoiesis, thus causing anemia, and may be an under-recognized anemia-related factor. Therefore, low BT may lead to a decrease in hemoglobin and HbA_1c_ levels [20].

Previous studies showed that serum BT was positively correlated with HbA_1c_ in men with type 2 diabetes [10], but this population had been treated with hypoglycemic drugs. One study also found that obese men with type 2 diabetes had lower testosterone levels than those with normal glucose tolerance [21]. Here, we selected the non-diabetic population to investigate whether sex hormones, including BT, were associated with HbA_1c_ levels in these individuals. We found that serum BT and HbA_1c_ were marginally correlated in men; while after adjusting for other factors, BT was correlated with other sex hormones, but no correlation was found between BT and HbA_1c_. Additionally, we found that E_2_ was positively correlated with HbA_1c_ in men. Mean age of men included in this study was 60.3 years old. Moreover, testosterone levels gradually decrease with age [19], while the activity of aromatase increases, which activates the conversion of testosterone to estradiol [22, 23]. Thus, the above results may be related to the ability of aromatase to convert testosterone into estradiol.

We also found that SHBG was negatively correlated with HbA_1c_ in postmenopausal women. Previous study showed a significantly negative correlation between SHBG and HbA_1c_ in non-diabetic postmenopausal women (*n* = 200) after adjusting for age and BMI [3]. In this study, data of a large number of postmenopausal women was further adjusted for other metabolic factors. SHBG is synthesized and secreted by the liver cells to primarily combine with sex hormones and regulate their physiological effects [24]. Testosterone stimulates erythropoiesis, and while its levels are significantly reduced in postmenopausal women, the change in SHBG is not obvious [25]. The affinity of SHBG for androgens is much greater than that for estrogen, and while circulating, SHBG concentration is easily affected by the activity of peripheral androgens. It is regarded as one of the biological effects of androgens [26]. This may explain why SHBG is related to HbA_1c_. Additionally, we found that hemoglobin was a negative factor, while BMI was a positive factor for HbA_1c_ in men. Age was a positive factor for HbA_1c_, in both men and postmenopausal women, consistent with results of previous studies [27, 28].

This study has some limitations. First, the study population only included middle-aged and elderly individuals, and hence, the results may not be generalized to entire community. Second, the cross–sectional study design could not determine the causal relationship between changes in sex hormone and HbA_1c_ levels. Therefore, prospective studies with a larger sample size and different age groups are needed to validate these findings.

In summary, sex hormones influence HbA_1c_ levels in non-diabetic population. Moreover, levels of E_2_ were independently and positively correlated with HbA_1c_ in men, and those of SHBG were independently and inversely correlated with HbA_1c_ in postmenopausal women.

## Supplementary Information

**Additional file 1.**

## Data Availability

The data used to support the findings of this study are available from the corresponding author upon request.
