# Supplementary material for: The relationship between sex hormones and glycated hemoglobin in a non-diabetic middle-aged and elderly population
Source: BMC Endocr Disord. 2022 Apr 5;22:91. doi: 10.1186/s12902-022-01002-w (PMC8985254; doi:10.1186/s12902-022-01002-w)
Supplement: Supplementary file 1 — Additional file 1. [file 12902_2022_1002_MOESM1_ESM.doc]

**Date of investigation: ID:**

**Name: Gender: Date of birth:**

**Marital status: Phone number:**

**Education: Income: Occupation:**

**Do you smoke?-**

○Current, average cigarettes smoked per day ○Used to ○Never

**Do you drink?-**

○Current, type of wine Frequency of drinking

○Used to

○Never

**In the past 7 days, how many days did you perform high physical activity, such as lifting (lifting) heavy objects, running, swimming, aerobics in the gym, etc.?**

_____day a week; on these days, averaged _____ hours ____ minutes of these high physical activities per day

**In the past 7 days, how many days did you engage in moderate-intensity physical activity, such as lifting light objects, cycling, playing table tennis or badminton, ballroom dancing, etc.?**

_____day a week; on these days, averaged _____ hours ____ minutes of these moderate physical activities per day

**In the past 7 days, how many days have you walked more than 10 minutes a day?**

_____day a week; on these days, averaged _____ hours ____ minutes per day of walking

**Have you used steroids hormones in the past year?** Yes No

**Are you in menopausal states?**  Yes No

If menopause, time of menopause ; Are you receiving hormone replacement therapy? Yes No

**What is your menarcheal age?**

**How many times have you given birth?**

**Do you have the following diseases and medication history?**

| Diseases | Diagnosis time | Medical treatement | Others |
| --- | --- | --- | --- |
| Diabetes |  |  | Symptom: Glucose: |
| Hypertension |  |  | Blood pressure: |
| Dyslipidemia |  |  | TC: TG: HDL-c: LDL-c: |
| Angina pectoris |  |  | Coronary intervention: Coronary bypass: |
| Myocardial infarction |  |  | Coronary intervention: Coronary bypass: |
| Heart failure |  |  | / |
| Stroke |  |  | Cerebrovascular intervention: |
| Carotid plaque |  |  | / |
| Lower extremity vascular disease |  |  | / |
| Renal dysfunction |  |  | / |
| Gout |  |  | / |
| Hyperthyroidism |  |  | Thyroid hormone: |
| Hypothyroidism |  |  | Thyroid hormone: |
| Cancer |  |  | Classess: |

**Family history (If yes, please fill in the number)**

|  | Father | Mother | Brothers and Sisters | Children |
| --- | --- | --- | --- | --- |
| Hypertension |  |  |  |  |
| Diabetes |  |  |  |  |
| Dyslipidemia |  |  |  |  |
| Stroke |  |  |  |  |
| Coronary heart disease |  |  |  |  |
| Gout |  |  |  |  |
| Obesity |  |  |  |  |
| Myocardial infarction |  |  |  |  |
